# Scalp Metastasis as an Initial Manifestation of Squamous Cell Carcinoma of the Lung: Case Report of an Extremely Rare Entity

**DOI:** 10.7759/cureus.35074

**Published:** 2023-02-16

**Authors:** Muhammad Tahir, Kurt Knowles, Elba Turbat-Herrera, John J Lazarchick, Joe S Liles

**Affiliations:** 1 Pathology and Laboratory Medicine, University of South Alabama Hospital, Mobile, USA; 2 Pathology and Laboratory Medicine, Mobile Infirmary Medical Center, Mobile, USA; 3 Surgery, University of South Alabama Health System, Mobile, USA

**Keywords:** lung cancer, cutaneous metastasis, scalp metastasis, non small cell lung cancer, squamous cells carcinoma

## Abstract

Cutaneous metastasis of primary visceral neoplasm is an unusual phenomenon. However, cutaneous metastasis as an initial presentation of clinically silent visceral neoplasm is exceedingly rare. We are reporting a unique case of an elderly male patient who presented with a solitary scalp metastasis as an initial manifestation of underlying lung cancer. Further diagnostic evaluation revealed neoplastic primary lung disease.

This case report emphasizes the importance of physicians being aware of these unusual clinical presentations of visceral malignancies. It is also critical to order appropriate diagnostic tests promptly to establish an accurate diagnosis and begin the proper treatment for a better prognosis. Skin lesions can be a diagnostic manifestation of lung cancer and predict a poor prognosis.

We conclude that in patients with a history of smoking or lung cancer who present with cutaneous lesions, the possibility of skin metastasis of primary lung cancer should always be considered in the differential diagnosis.

## Introduction

Overall incidence of cutaneous metastases from primary visceral tumors ranges from 0.2% to 10% [1]. This could occur as a consequence of direct tumor invasion as a local metastasis or distant metastasis. Oftentimes, cutaneous metastases appear later in the course of the disease and after the initial diagnosis of the primary visceral malignancy [2]. In extremely rare circumstances, it can happen simultaneously at the time the primary malignancy has been diagnosed. A skin metastasis from lung cancer is an uncommon clinical condition that has been determined to affect 0.22-12% of lung cancer patients [1-3]. It is extremely uncommon for skin metastases to be the first and only sign of an underlying lung malignancy [4].

The chest, abdomen, head, and neck are the most common sites where lung cancer-related skin metastases most frequently occur. Rarely, it may manifest as a single lesion on the scalp [5]. When compared to other cancers, lung cancer metastasis to the skin occurs more quickly after its initial diagnosis. Grossly, a cutaneous metastasis of lung is indistinguishable from other metastatic visceral malignancies. Cutaneous metastasis of lung should be considered in the differential diagnosis among patients who present with cutaneous nodular lesions [6]. Patients with risk factors for lung cancer such as smoking should be heavily investigated with proper diagnostic modalities for cutaneous metastases. The median survival period following the diagnosis of the cutaneous metastases is between 2.9 and 4.9 months, with a very dismal prognosis [7].

Here, we report and discuss an exceedingly rare case of an elderly asymptomatic male patient who presented with a solitary scalp lesion as an initial presentation of an underlying squamous cell carcinoma of lung.

## Case presentation

**Figure 1:**
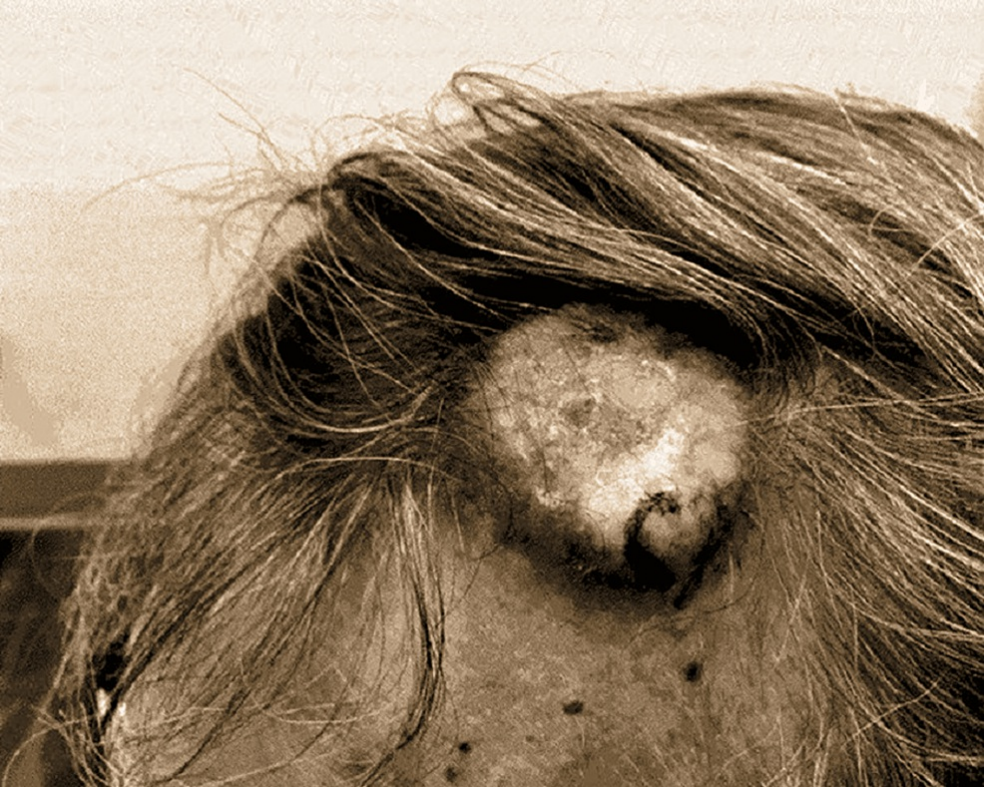
Large nodular scalp lesion with crusting and focal necrosis.

A 74-year-old male patient with high blood pressure, elevated cholesterol, tobacco smoking, and prostate cancer developed a skin lesion on the left frontal scalp. The mass was reported to be pea-sized approximately four months before presentation and had rapidly increased in size. Grossly, at the time of presentation, the lesion was nodular, crusted with focal erythema and ulceration as seen here (Figure 1).

**Figure 2:**
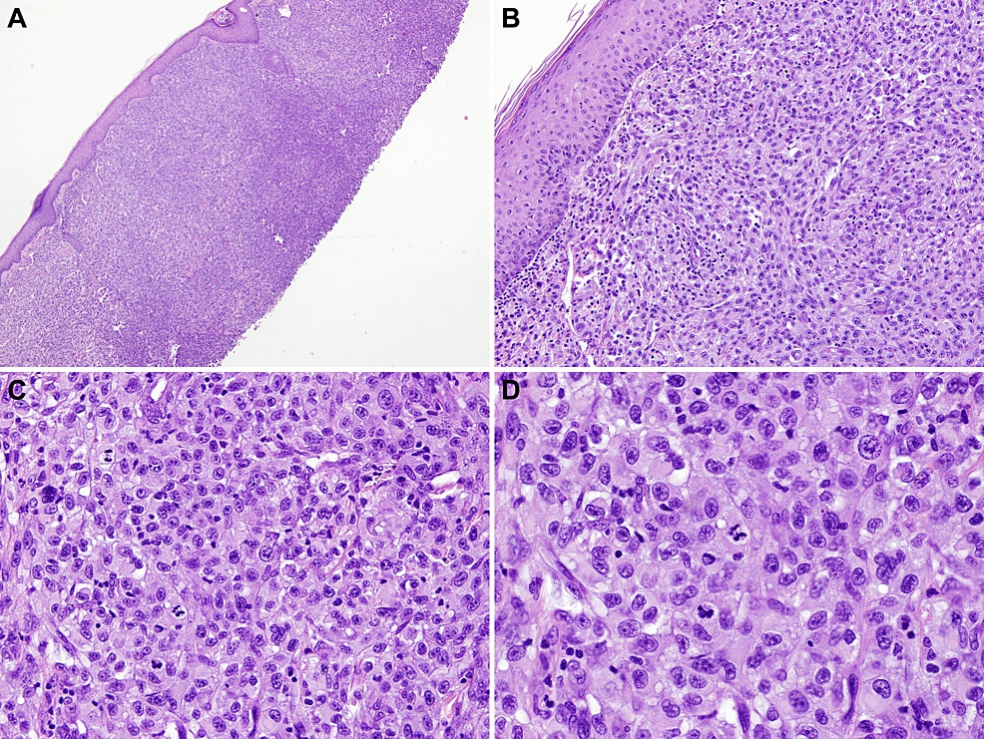
Low power view (4X, 10X) showing atypical neoplastic cells in the dermis with overlying epidermis without any atypia (A and B). Medium power view (40X) showing poorly differentiated, pleomorphic atypical neoplastic cells population (C). High power view (60X) showing neoplastic cells with irregular nuclear contour, dispersed granular chromatin and atypical mitotic figures (D).

A biopsy was done, and histology showed a spindle cell squamous cell. Carcinoma diagnosis was rendered by the outside dermatology laboratory. Later, the case was sent to us at the University of South Alabama Health Hospital for consultation. Histologically, the tumor was dermal-based and composed of very atypical squamous cells with no connection to the overlying epidermis. There were abundant atypical mitoses, dyskeratotic keratinocytes and apoptotic bodies seen on Hematoxylin and Eosin (H&E) stains (Figure 2). The overlying epidermis showed no atypia.

**Figure 3:**
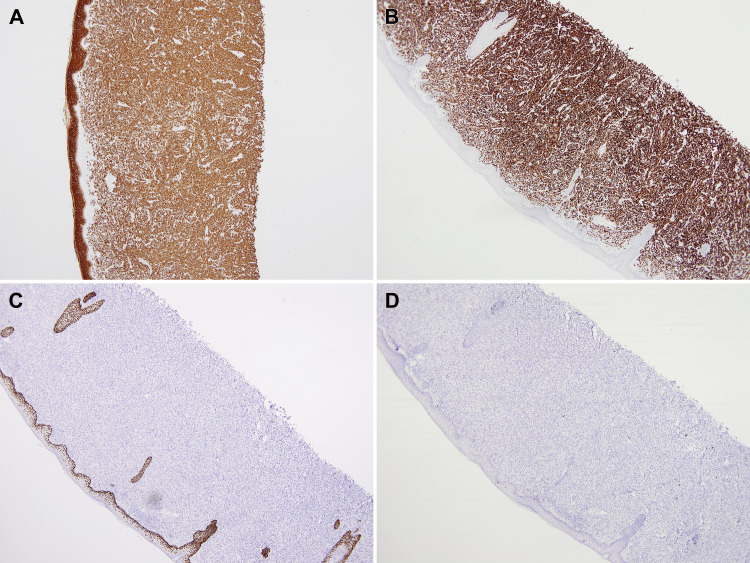
A: High molecular weight cytokeratin (HMWCK) positive in overlying epithelium and dermal neoplastic cells (4X). B: Cytokeratin-7 (CK-7) positive in neoplastic cells (4X). C: P63 positive in epithelium and negative in neoplastic cells (4X). D: Negative cytokeratin-20 (CK-20) in both epithelium and neoplastic cells (4X). P63: tumor protein 63, a transcription factor of the p53 gene family

Immunohistochemically, the tumor cells were positive for high molecular weight cytokeratin (HMWCK), cytokeratin-7 (CK7), and negative for cytokeratin-20 (CK20), ruling out primary squamous cell carcinoma of skin (Figure 3).

**Figure 4:**
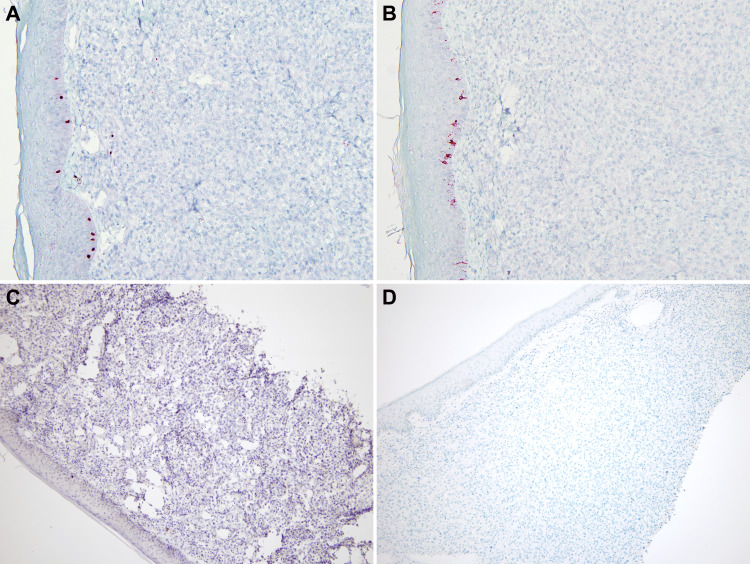
A and B: Negative melanin A and SOX-10 stain in neoplastic cells with focal positivity in normal melanocytes at dermal epidermal junction (4X). C and D: Negative PSA and NKX3.1 stain (4X). SOX-10: Sex-determining region Y-related high mobility group-box 10, PSA: Prostate specific antigen, NKX3.1: Prostatic tumor suppressor gene

**Figure 5:**
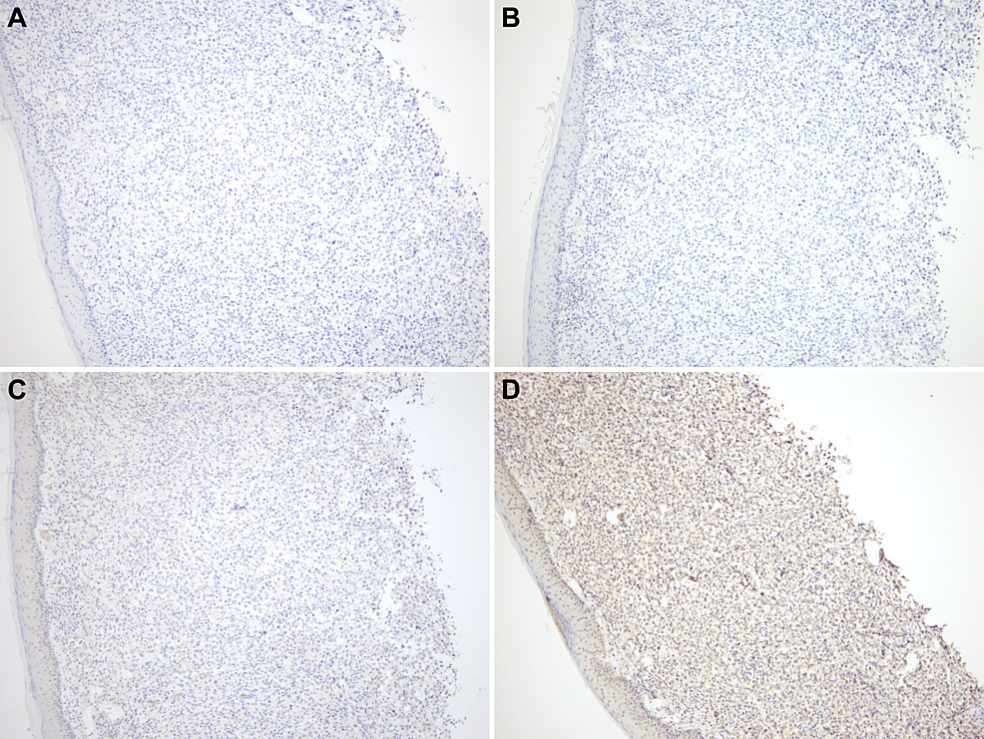
A and B: Negative CD-56 and synaptophysin (4X). C and D: Negative napsin A and TTF-1 (4X). CD-56: Neural cell adhesion molecule, TTF-1: Thyroid transcription factor 1

Tumor cells were negative for prostate-specific antigen (PSA), NKX3.1, melanin A, SOX-10, CD-56, synaptophysin, napsin A, and TTF1, ruling out the possibility of metastatic prostate cancer, melanoma, neuroendocrine tumor, and primary lung adenocarcinoma respectively (Figures 4, 5). Based on the immunohistochemical profile a diagnosis of poorly differentiated carcinoma most likely representing a squamous cell metastasis from the lung was made.

**Figure 6:**
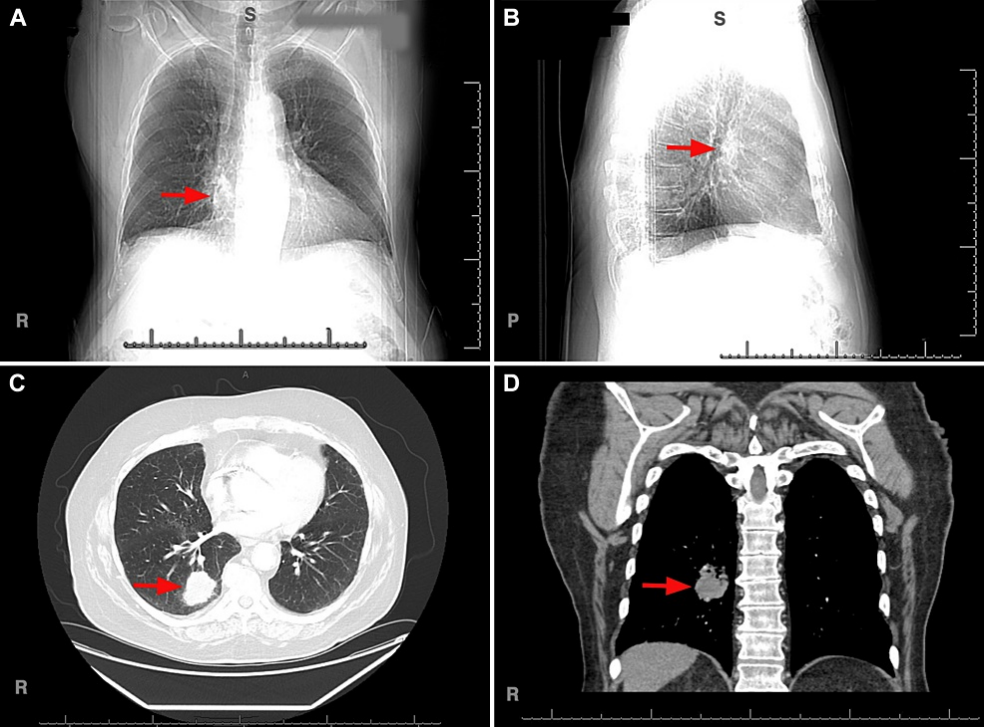
Chest x-ray anterior and posterior view, red arrows indicating the lesion (A and B). CT scan with contrast, red arrows indicating the lesion (C and D).

Total body scan was recommended to look for the primary origin of the tumor. On anterior and posterior chest x-ray and Computed Tomography (CT) scan of the chest, a 6 cm right lower lobe mass extending into the hilum, highly suspicious for primary pulmonary malignancy was identified (Figure 6).

**Figure 7:**
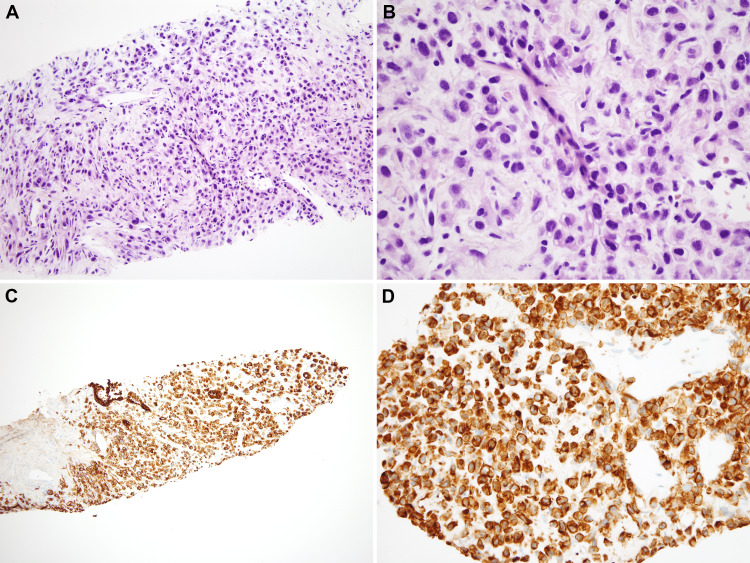
A: Low power view (10X) showing the sheets of atypical neoplastic cells. B: High power view (60X) showing neoplastic cells with nuclear atypia, dispersed granular chromatin and atypical mitotic figures. C and D: Low power (4X) and high power (20X) view showing diffuse positivity of neoplastic for pan-cytokeratin.

Patient had a bronchoscopy performed and cytological examination only showed atypical cells but not diagnostic of lung cancer. Subsequent CT-guided biopsy showed a poorly differentiated carcinoma confirming primary lung origin (Figure 7). 

## Discussion

Tumors originating from the scalp are rare neoplasms accounting for almost 2% of all skin cancers. They can arise from epidermis, dermis, and pilosebaceous units or present as a metastasis from other cancers. The scalp being rich in blood supply and lymphatics makes it a favorite site for metastases of visceral malignancies accounting for 4 to 6.9% of the cutaneous metastatic cancers [2,8]. The most common histological subtypes of cancers affecting the scalp are squamous cell carcinomas and basal cell carcinomas as reported by Chiu et al. [9]. Metastatic tumors to the scalp are reported in 12.8% of the cases and lung is the most common primary tumor. Scalp metastasis as an initial presentation of an underlying lung cancer is an exceedingly rare clinical scenario and not frequently seen or reported in the literature [1].

The clinical manifestation of cutaneous metastases varies. Cutaneous metastasis of different subtypes of lung cancers usually occurs in chest wall, abdomen, back and upper extremities. The most typical sign is a solitary lesion or numerous nodules, however inflammatory and a sclerodermoid shape can also be present. Additionally, lesions can mimic other conditions such erythema annulare, condyloma, epidermal inclusion cysts, and herpes zoster infection [10]. Most lesions have a diameter of 1 to 5 cm and a variety of physical features. Nodular lesions can be firm, immobile, painful, ulcerated with induration, and can extend deep into the dermis and the subcutaneous tissues. In our case, the lesion started as a tiny scalp nodule that was rapidly growing with crusted, ulcerated overlying skin [2].

Skin metastasis from visceral malignancies tends to occur in close proximity to the primary neoplasm by direct extension and contiguous tissue infiltration or through blood or lymphatic spread. In our case of an advanced large lung mass, the lymphatic and hematogenous spread led to skin metastasis [11]. In some situations where ulcerated lesions are present, metastatic skin cancers cannot be distinguished from primary skin malignancies mainly on the basis of clinical characteristics. The clinical manifestation of primary skin cancer is variable and depends on the tumor size, color, consistency, and type of neoplasm. Therefore, the histologic examination is the best way to distinguish a metastatic skin lesion from a primary tumor [2,7].

In order to make a diagnosis of a cutaneous metastasis, biopsy of the lesion is the gold standard diagnostic procedure. Histopathological analysis, a history of metastatic skin lesions, and a clinical diagnosis of malignancy are required in clinical practice for a final diagnosis of skin metastasis. Moderately to poorly differentiated adenocarcinoma of the lung is the most typical lesion that leads to cutaneous metastasis [8]. Immunohistochemical (IHC) markers are vital for a determination of a primary cancer. In a patient with no prior history of malignancy the histological diagnosis of primary cancer may be incredibly challenging. However, particularly for elderly individuals who present with solitary or multiple cutaneous lesions, these should be carefully evaluated since these lesions may be the indicators of an underlying visceral malignancy [12].

Treatment of cutaneous metastasis is focused on the treatment of the primary malignancy that can be achieved clinically by surgical resection and the combination of adjuvant radiation and chemotherapy. In general, the patient with cutaneous metastases of lung cancer usually presents in advanced stage and only palliative treatment is preferable. In most cases, the patients have widespread advanced metastatic disease affecting bones, liver, brain, and or lungs. Because of advanced disease the prognosis of these patients is poor, with reported median survival of three to six months after diagnosis [13,14]. A small number of cases with slightly favorable prognosis has been reported in a patient with skin metastasis who had no other distant lesions. In a few instances, the scalp metastasis can spontaneously regress after treatment of the primary cancer as reported by Miyazaki et al. [15]. In general, regardless of the type of primary malignancy or site of metastasis, the dissemination of an internal malignancy to the skin is an ominous sign indicating a poor prognosis with imminent death [7].

## Conclusions

Solitary scalp metastasis of squamous cell carcinoma of lung is an exceedingly rare presentation. Despite its rarity, patients with history of smoking and lung cancer should be carefully examined for the skin lesions and metastatic skin disease should always be considered in the differential diagnosis. We report this case to provide awareness that clinicians should be aware of the existence of this rare entity, characteristic features, and the prognostic significance of cutaneous metastasis of lung cancer. We recommend early recognition, staging and initiation of appropriate treatment for a better outcome. Furthermore, we emphasize that careful histologic examination of skin lesions is particularly important because it can provide valuable clues to the occult internal malignancies.
